# Effect of Exercise and Omega-3 Supplements on the Quality of Life of Young Female Patients With Primary Dysmenorrhea: A Randomized Controlled Trial

**DOI:** 10.7759/cureus.88044

**Published:** 2025-07-15

**Authors:** Renu Kumari, Munish Rastogi, Dolly Rastogi, Samarjeet Kaur, Atosh Kumar, Preeti Kanawjia, Jayvardhan Singh

**Affiliations:** 1 Physiology, Ganesh Shankar Vidyarthi Memorial Medical College, Kanpur, IND; 2 Medical Microbiology, Institute of Health Sciences, Chhatrapati Shahu Ji Maharaj University, Kanpur, IND; 3 Community Medicine, Ganesh Shankar Vidyarthi Memorial Medical College, Kanpur, IND

**Keywords:** aerobic exercise, dysmenorrhea, fatty acid, menstrual pain, omega-3 supplements

## Abstract

Background

Primary dysmenorrhea, prevalent among women of reproductive age, manifests as menstrual pain and discomfort localized in the lower abdomen. This study aimed to observe the effect of exercise, omega-3 fatty acids, and the clubbing of exercise and omega-3 supplements on primary dysmenorrhea.

Methods

A randomized controlled trial was conducted involving young female patients. We assessed a cohort of 72 female patients, aged 18 to 25 years, to determine the effects of exercise and omega-3 fatty acids on primary dysmenorrhea. Parameters such as visual analog scale (VAS) scores, erythrocyte sedimentation rate (ESR), C-reactive protein (CRP), and complete blood count (CBC) parameters were evaluated before and after the intervention.

Results

At baseline, no statistically significant differences were detected among the four groups (control, exercise, omega-3, and combined exercise plus omega-3) regarding VAS scores (p=0.605), CRP levels (p=0.164), hemoglobin levels (p=0.104), or platelet counts (p=0.949). Nevertheless, significant differences were identified in ESR (p=0.002) and total leukocyte count (TLC) (p<0.001). The omega-3 group exhibited elevated levels of ESR and TLC, particularly in conjunction with the combined exercise and omega-3 intervention. Following 12 weeks of intervention, notable enhancements were recorded across various parameters. The VAS scores demonstrated a statistically significant reduction across all groups (p=0.023), with the most pronounced alleviation observed in the cohort receiving both exercise and omega-3 supplementation, thereby suggesting an enhanced efficacy in pain relief. Additionally, CRP levels revealed significant intergroup variances (p=0.016), with the exercise group exhibiting the lowest recorded levels. The TLC remained significantly different between the groups (p<0.001), with consistently elevated levels in the omega-3 group and diminished levels in the combined intervention group. Conversely, no statistically significant alterations were detected in ESR (p=0.086), hemoglobin (p=0.077), or platelet counts (p=0.871) at the conclusion of the 12-week evaluation.

Conclusion

A notable reduction in the intensity of menstrual pain, as quantified by the VAS, an indicator of systemic inflammation, was observed with omega-3 supplementation and exercise. Moreover, the reduction in the ESR and CRP levels also implied that the implemented interventions may have facilitated an anti-inflammatory response.

## Introduction

Dysmenorrhea, derived from the Greek terms dys (difficult), mens (month), and rhoia (flow), denotes the phenomenon of painful or challenging menstrual flow [1]. Dysmenorrhea encapsulates the subjective experience of painful menstruation in females. The aim of the present randomized controlled trial was to evaluate the effect of exercise and omega-3 fatty acid supplementation on the quality of life of young female patients with primary dysmenorrhea. The specific objective was to assess and compare the individual and combined effects of exercise and omega-3 supplementation on the severity of dysmenorrhea and associated symptoms. Typically, dysmenorrhea is classified into two distinct categories: primary dysmenorrhea, which manifests in the absence of any underlying pelvic pathology, and secondary dysmenorrhea, which is linked to identifiable medical conditions that affect the reproductive system [2].

Primary dysmenorrhea is characterized by recurrent menstrual discomfort that is not associated with any identifiable pelvic pathology [3]. This condition exhibits a high prevalence, with approximately 21% to 26% of women experiencing severe menstrual discomfort [4]. In addition to the pain, individuals afflicted with primary dysmenorrhea commonly report disturbances in sleep patterns and a reduction in overall quality of life. Severe menstrual pain may also result in substantial economic ramifications, including increased absenteeism from employment, diminished work productivity, and a two-to-threefold escalation in healthcare expenditure [3,5]. Research has demonstrated that during the secretory phase of the menstrual cycle, progesterone exerts a crucial anti-inflammatory function by inhibiting the activation and release of metalloproteinases. Progesterone is also instrumental in modulating the synthesis of prostaglandins and leukocytes. The predominant factor contributing to discomfort in dysmenorrhea is the elevated concentration of prostaglandins within the organism [6]. Due to the exorbitant expense, potential adverse effects, and contraindications linked to certain pharmaceutical interventions, coupled with the increasing availability and public interest in complementary therapies, a plethora of investigations are examining alternative approaches to the management of dysmenorrhea. In the past two to three decades, consistent exercise and physical activity have been acknowledged as efficacious strategies for both the prevention and management of dysmenorrhea.

Physical activity encompasses any bodily movement produced by skeletal muscles that culminates in energy expenditure [7]. Conversely, physical fitness pertains to a collection of attributes that individuals possess or cultivate, which are associated with their capacity to execute physical activity proficiently [8]. Aerobic exercise has been demonstrated to mitigate the detrimental effects of dysmenorrhea on the quality of life and social functioning of women. Women who partake in regular aerobic activities frequently report diminished physical symptoms and discomfort throughout their menstrual cycle [9]. Healthcare professionals frequently advocate for aerobic exercises such as pelvic tilts, walking, cycling, and swimming, as these pursuits can enhance blood circulation, relax abdominal musculature, alleviate pelvic pain, and relieve pressure on nerve centers, pelvic organs, and the gastrointestinal tract. Consumption of foods rich in omega-3 fatty acids diminishes the likelihood of experiencing pain associated with dysmenorrhea. According to a systematic review, omega-3 fatty acids assist in inhibiting the synthesis of arachidonic acid, which subsequently curtails the production of prostaglandins, ultimately resulting in a reduction of myometrial and vascular contractions [10]. During menstruation, a decline in progesterone levels instigates the synthesis of arachidonic acid. This acid, in conjunction with cyclooxygenase enzymes, facilitates the production of series 2 prostaglandins, which are pro-inflammatory and contribute to uterine discomfort. Upon consumption of omega-3 fatty acids, they serve as a substrate for cyclooxygenase enzymes, thereby promoting the synthesis of series 3 prostaglandins. Consequently, the concentration of pro-inflammatory series 2 prostaglandins diminishes, while the levels of anti-inflammatory series 3 prostaglandins increase. Prostaglandin series 3 aids in diminishing myometrial contractions, thereby alleviating ischemia and discomfort during menstruation [11].

## Materials and methods

**Figure 1:**
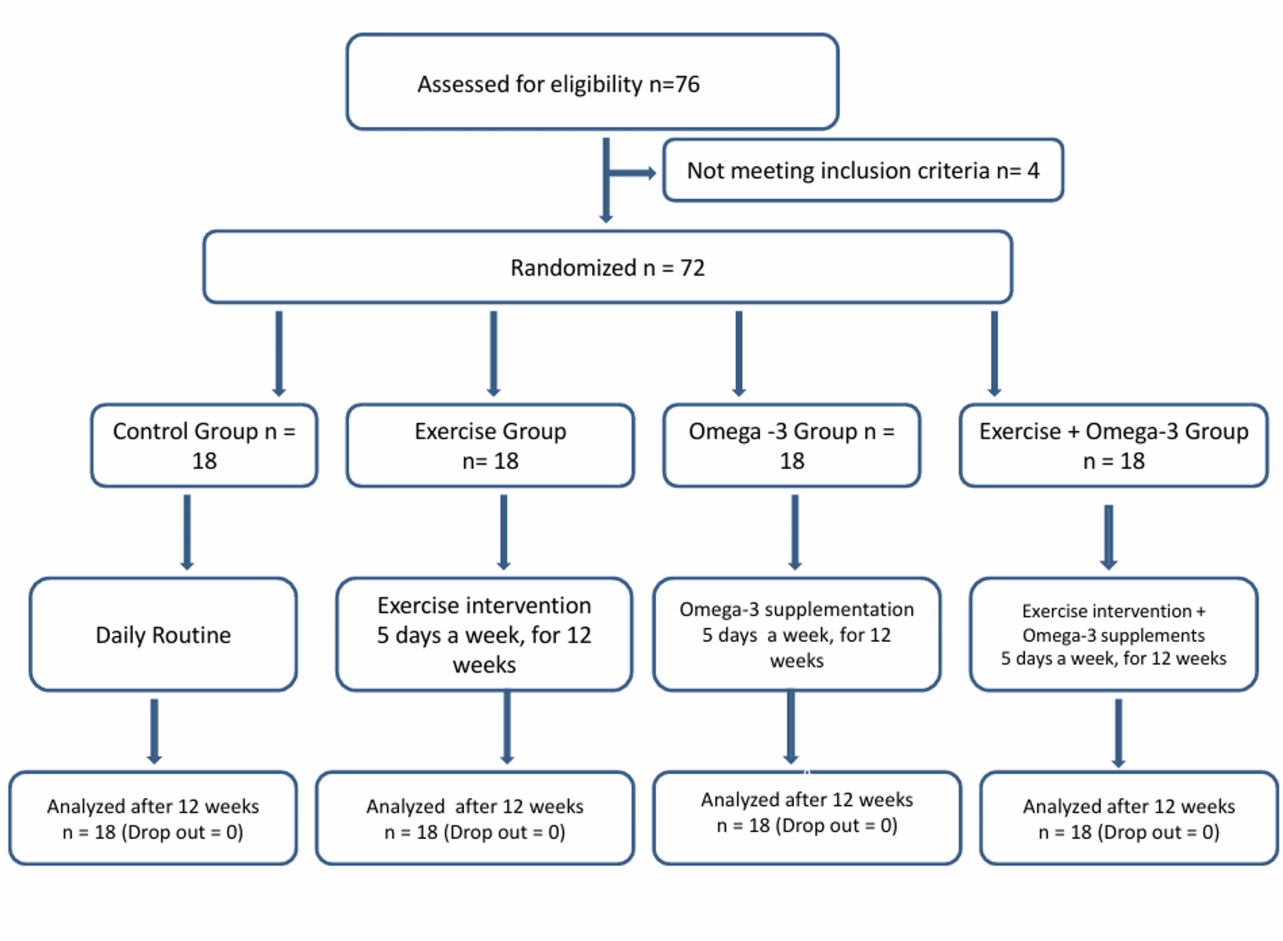
CONSORT diagram CONSORT: Consolidated Standards of Reporting Trials [13]

A randomized controlled trial was carried out from November 2024 to May 2025 at Lala Lajpat Rai and associated hospitals, Ganesh Shankar Vidyarthi Memorial Medical College, Kanpur, a tertiary care hospital in North India, involving 72 young female patients diagnosed with primary dysmenorrhea. The sample size for evaluating the effects of exercise and omega-3 supplementation on this population was determined using OpenEpi software [12] using the module for comparing means and standard deviation of two independent samples. Based on these parameters, the minimum required sample size was calculated to be 68 participants (17 per group). Participants were randomly allocated to one of four groups: (1) Control, (2) Exercise, (3) Omega-3 supplementation, and (4) Combined exercise and omega-3 supplementation group. Randomization was performed using a computer-generated random number table. A block randomization method with varying block sizes (four and eight) ensured balanced distribution across groups while maintaining allocation unpredictability. To account for potential dropouts, a total of 72 participants were enrolled in the study. This decision was based on previous studies in similar settings, which have reported dropout rates ranging from 10% to 20%, primarily due to non-compliance, scheduling conflicts, or loss to follow-up. The Consolidated Standards of Reporting Trials (CONSORT) diagram is shown in Figure 1 [13].

Before the study commenced the diagnosis of dysmenorrhea was made by consulting a gynecologist, who evaluated the patient based on their medical history and the Menstrual Symptom Questionnaire (MSQ) Table 1 [14].

**Table 1:** Menstrual Symptom Questionnaire (MSQ)

S.N.	ITEM	NEVER 1	RARELY 2	SOMETIME 3	OFTEN 4	ALWAYS 5
1.	I feel irritable, easily agitated & I am impatient a few days before my period.	N	R	S	O	A
2.	I have cramps that begin on the first day of my period.	N	R	S	O	A
3.	I feel depressed for several days before my period.	N	R	S	O	A
4.	I have abdominal pain or discomfort which begins one day before my period.	N	R	S	O	A
5.	For several days before my period I feel exhausted, lethargic or tired	N	R	S	O	A
6.	I only know that my period is coming by looking at the calendar.	N	R	S	O	A
7.	I take a prescription drug for the pain during my period.	N	R	S	O	A
8.	I feel week & dizzy during my period.	N	R	S	O	A
9.	I feel tense & nervous before my period.	N	R	S	O	A
10.	I have diarrhea during my period.	N	R	S	O	A
11.	I have backaches several days before my period.	N	R	S	O	A
12.	I take aspirin for the pain during my period.	N	R	S	O	A
13.	My breast feels tender & sore a few days before my period.	N	R	S	O	A
14.	My lower back, abdomen, & the inner sides of my thighs begin to hurt or be tender on the first day of my period.	N	R	S	O	A
15.	During the first day or so of my period, I feel like curling up in bed, using a hot water bottle on my abdomen, or taking a hot bath.	N	R	S	O	A
16.	I gain weight before my period.	N	R	S	O	A
17.	I am constipated during my period.	N	R	S	O	A
18.	Beginning on the first day of my period, I have pain which may diminish or disappear for several for several minutes & then reappear.	N	R	S	O	A

19.	The pain I have with my period is not intense, but a continuous dull aching.	N	R	S	O	A
20.	I have abdominal discomfort for more than one day before my period.	N	R	S	O	A
21.	I have backaches which begin the same day as my period.	N	R	S	O	A
22.	My abdominal area feels bloated for a few days before my period.	N	R	S	O	A
23.	I feel nauseous during the first day or so of my period.	N	R	S	O	A
24.	I have headaches for a few days before my period.	N	R	S	O	A
25.	TYPE 1 The pain begins on first day of menstruation, often coming within an hour of the first signs of menstruation. The pain is most severe the first day & may or may not continue on subsequent days. Felt as spasms, the pain may lessen or subside for a while and then reappear. The pain is so severe to cause vomiting, fainting or dizziness. Feel most comfortable in bed or taking a hot bath. This pain is limited to lower abdomen, back & inner sides of thighs.	Type 1				
	TYPE 2 At the beginning there is increasing heaviness, and a dull aching pain in the lower abdomen. The pain is sometimes accompanied by nausea, lack of appetite & constipation. Headache, Backache & Breast pain are also present sometimes.	Type 2				

A thorough medical history was taken for each participant to exclude any underlying pathological conditions. All participants received a clear and concise explanation of the exercise regimen and its potential benefits, fostering trust and encouraging active participation throughout the study period. Written informed consent was obtained from each participant prior to their inclusion in the research.

The study was approved by the Ethics Committee, Ganesh Shankar Vidyarthi Memorial Medical College (approval no. EC/BMHR/2023/73 dated 20-10-2023). The trial was prospectively registered in Clinical Trials Registry- India (CTRI; study no. CTRI/2024/11/076771). The inclusion criteria were subjects with primary dysmenorrhea, age group between 18-25 years, and those with regular menstrual cycles. Secondary dysmenorrhea was ruled out via ultrasound. The exclusion criteria were any medical or gynecological history suggestive of secondary dysmenorrhea, married female patients, and those with a history of pelvic surgery.

Measures

Visual Analog Scale (VAS)

**Figure 2:**
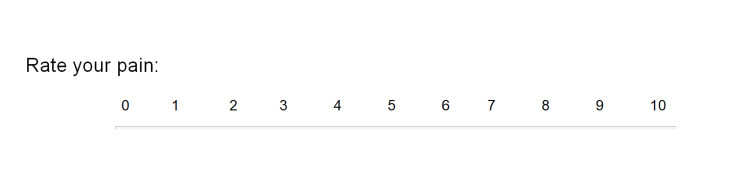
Traditional paper-based visual analog scale (VAS) for adults

The VAS was employed to assess the intensity of menstrual pain both at baseline and following the intervention. This numerical scale ranges from 0 to 10, where 0 indicates "no pain" and 10 signifies the "worst imaginable pain" (Figure 2) [15].

Biochemical Analyses

Overnight fasting venous blood samples (5 ml) were collected both before and after 12 weeks of intervention. All the tests were conducted during the follicular phase of the menstrual cycle. Samples were obtained after a minimum of 12 hours of overnight fasting from each participant.

Intervention

Control Group

The control group was observed throughout the study period without intervention. They did not participate in a formal exercise program, and did not take any chemical or herbal drugs or supplements to prevent their dysmenorrhea during their menstrual period. Also, they did not have any lifestyle modifications.

Aerobic Exercise Group

Moderate exercise was performed over a 12-week period, consisting of five sessions per week, each lasting 45 minutes, with the goal of achieving an approximate 50% increase in heart rate from its pre-exercise level, excluding the days of menstruation. Each exercise stage included: Warming movements for 10 minutes (head movements, shoulder rotation, and balance). The subjects were allowed to rest for five minutes while seated in a chair, during which their resting pulse rate was measured. The procedure for the modified Harvard step test [16] was then thoroughly explained to them. To ensure clarity and reduce any anxiety, the test was demonstrated before the subjects were instructed to perform it in a rhythmic manner. The recorded data were collected accordingly.

Omega-3 Supplementation Group

Eight hundred milligrams of omega-3 capsules (Eicosapentaenoic acid (EPA) and Docosahexaenoic acid (DHA)) were administered five days a week for a period of 12 weeks. The participants did not change their usual diet and physical activity throughout the investigation.

Combined Exercise and Omega-3 Supplement Group

Exercises were done over a period of 12 weeks, at five sessions in a week with 45 min for each session excluding days of menstruation and 800 milligrams of omega-3 capsules were administered five days a week for a period of 12 weeks.

Statistical analysis

The data obtained was tabulated in an MS Excel worksheet (Microsoft Corp., Redmond, WA, US) and analyzed by using R Software (R Foundation for Statistical Computing, Vienna, Austria). Continuous variables are expressed as mean ± standard deviation. One way ANOVA test was used to compare the means of the four groups (control group, aerobic exercise group, omega-3 supplementation group, and combined exercise and omega-3 supplement group) to determine if a statistically significant difference existed between them, and the Tukey post-hoc test was applied wherever relevant. A p-value of <0.05 was considered to indicate statistical significance. 

## Results

This study was conducted on 72 subjects and included cases of primary dysmenorrhea. The subjects were divided into four groups: control group (n=18), exercise group (n=18), omega-3 supplement group (n=18), and combined exercise + omega-3 supplement group (n=18). The parameters that we included in this study were height, weight, BMI as anthropometric parameters and inflammatory markers, i.e. erythrocyte sedimentation rate (ESR), CRP, and complete blood count (CBC) levels that included hemoglobin (Hb), total leucocyte count (TLC), and platelets.

Since it was a comparative study, we compared the role of various interventions among the four study groups. At baseline, there were no statistically significant differences among them in terms of VAS, CRP, hemoglobin, and platelet count (p>0.05), indicating that these variables were comparable across groups prior to intervention. However, ESR (p=0.002) and TLC (p <0.001) were significantly higher in the omega-3 and exercise groups, respectively (Table 2).

**Table 2:** Comparison of study variables at baseline The table compares the subjective pain experience and hematological parameters of participants of the four groups using One way ANOVA test at the start of the study. A p-value of <0.05 was considered statistically significant. VAS: Visual Analogue Scale; ESR: Erythrocyte Sedimentation Rate; CRP: C-Reactive Protein; Hb: Hemoglobin; TLC: Total Leucocyte Count; WBC: White Blood Cell

Study variable	Units	Mean/SD				p-value
		Control group (n=18)	Exercise group (n=18)	Omega-3 group (n=18)	Exercise+ Omega-3 group (n=18)	
VAS	score (0–10)	5.94/±1.68	5.88/±1.83	5.11/±2.17	5.56/±1.92	0.605
ESR	mm/hr	15.4/±7.01	24.6/±11.4	25.7/±11.4	25/±11.9	0.002
CRP	mg/L	2.48/±1.74	2.26/±1.67	4.41/±3.67	2.96/±2.29	0.164
Hb	mg/L	12.6/±1.26	13/±1.35	12.4/±1.14	13.4/±1.21	0.104
TLC	×10³ WBCs/µL	72.4/±10.8	75.5/±14.3	94.6/±16	69.5/±11.4	<0.001
Platelet count	×10³/µL	364/±82.7	374/±80.7	358/±84.2	370/±92	0.949

These baseline differences in inflammatory markers may introduce bias in interpreting the magnitude of post-intervention changes and should be considered as a limitation when assessing treatment effects. 

After 12 weeks of intervention, the VAS score was statistically significant and the p-value found to be 0.023. It was reduced in all the four groups and showed a >25% reduction in the combined intervention group, supporting its efficacy. The ESR after 12 weeks of intervention was reduced in exercise group followed by control group, combined exercise + omega-3 group, and then the omega-3 group. The CRP value was decreased in all the all groups and was statistically significant after 12 weeks, p-value found to be 0.016 and there was nearly equal significant association of CRP values between the exercise and omega-3 groups, although the lowest value was seen in exercise group. The difference in Hb was statistically non-significant in all the four groups before and after 12 weeks of intervention. TLC remained significantly different between groups (p<0.001), with persistently higher levels in the omega-3 group and reduced levels in the combined intervention group. There was no significant difference in platelet count across the four groups after 12 weeks of intervention (Table 3).

**Table 3:** Comparison of study variables after 12 weeks The table compares the subjective pain experience and hematological parameters of participants of the four groups using one way ANOVA test after 12 weeks of intervention. A p-value of <0.05 was considered statistically significant. VAS: Visual Analogue Scale; ESR: Erythrocyte Sedimentation Rate; CRP: C-Reactive Protein; Hb: Hemoglobin; TLC: Total Leucocyte Count; WBC= White Blood Cell

Study variable	Units	Mean/SD				p-value
		Control group (n=18)	Exercise group (n=18)	Omega-3 group (n=18)	Exercise + Omega-3 group (n=18)	
VAS	score (0–10)	5.47/±1.33	4.47/±1.50	4.11/±1.84	4.06/±1.47	0.023
ESR	mm/hr	13.5/±7.01	12.2/±6.46	18.3/±7.56	14.7/±8.65	0.086
CRP	mg/L	2.20/±2.06	0.864/±0.888	2.26/±1.99	1.03/±1.10	0.016
Hb	mg/L	12.6/±1.08	13.1/±1.08	12.9/±0.899	13.5/±0.917	0.077
TLC	×10³ WBCs/µL	73.8/±9.07	71.6/±14.4	90.5/±12.1	68.2/±10.8	<0.001
Platelet count	×10³/µL	365/±76.8	372/±76.8	352/±79.7	355/±85.6	0.871

## Discussion

In our study, we found that the intensity of pain, which was measured by the VAS, was high before intervention and reduced across all four groups after 12 weeks of intervention. The most significant decline occurred in the group receiving both exercise and omega-3 supplementation.

Exercise

Onur et al. investigated the influence of uncomplicated home-based exercises on dysmenorrhea and quality of life among a cohort of 40 women. The findings indicated a significant reduction in the intensity of dysmenorrhea, which persisted over the subsequent two months as well [17]. In contrast to these findings, Balkey et al. conducted an evaluation of menstrual pain and exercise levels among 654 university students and found no discernible correlation between exercise and primary dysmenorrhea [18]. Sutar et al. conducted a randomized clinical trial involving 100 female college students, who were allocated into "exercise" and "control" groups. Their results revealed a significant decline in pain (VAS scores) within the exercise group. The VAS scores exhibited a notable decrease immediately following the initiation of the exercise intervention and continued to diminish across the subsequent three assessments, achieving statistical significance (p<0.05) [19]. Jahromi et al. investigated the impact of weight training exercises on the characteristics of the menstrual cycle in a singular group of 250 students from Shiraz University, assessing conditions before and after the exercise regimen. Their research demonstrated a significant reduction in the intensity of dysmenorrhea post-intervention in comparison to baseline measurements [20]. Fallah et al. conducted a randomized clinical trial involving 70 students aged 15-18 years suffering from primary dysmenorrhea and discovered that engaging in regular physical activity resulted in a statistically significant alleviation of dysmenorrhea pain severity (p<0.05), pain rate index (PRI) (p<0.05), VAS (p<0.05), present pain intensity (PPI), total pain (TP) (p<0.05), and volume of bleeding (p<0.05) across the three groups studied [21]. Dehnavi et al. conducted a clinical trial involving 70 students with primary dysmenorrhea, randomly assigning participants to either intervention or control groups. Their findings indicated that the severity of primary dysmenorrhea at the commencement of the study did not exhibit a significant correlation between the exercise and control groups. In this study, after four weeks of intervention, no significant changes were observed in the intervention group compared to the control group (p=0.423), whereas, by the conclusion of eight weeks, the intervention group demonstrated significant improvements relative to the control group (p=0.041). Consequently, the outcomes of their investigation suggested that the implementation of aerobic exercise could enhance primary dysmenorrhea, and proposed aerobic exercise as a viable therapeutic option for this condition [22]. Noorbakhsh et al. documented that an eight-week regimen of physical activity led to a notable reduction in medication usage, as well as the amount and duration of bleeding, alongside a decrease in pain intensity among students experiencing primary dysmenorrhea [23]. These results substantiate our study, as the VAS scores exhibited a decline following a 12-week intervention across all four groups. Collectively, the evidence supports exercise as a first‑line, non‑pharmacologic approach to dysmenorrhea management. 

Omega‑3 supplementation

In the investigation conducted by Rahbar et al., female participants allocated to group 1 (n=47) were administered one omega-3 capsule daily for three months, followed by a placebo for another three months. Conversely, participants in group 2 (n=48) received a placebo for three months, followed by omega-3 supplementation for an equivalent duration. Their research revealed a significant reduction in pain intensity after three months of omega-3 fatty acid treatment (P<0.05) in group 1. Furthermore, the women who ingested omega-3 fatty acids required fewer rescue doses compared to those who received the placebo (P<0.05) [24]. Results of this study suggest that the use of omega-3 supplements can be effective in reducing symptoms such as pain, the need for analgesics, and inflammatory markers.

Combination of exercise and omega‑3 supplementation

In a randomized controlled trial conducted by Kumar et al., students from two medical institutions were selected and randomly assigned to three groups, A, B, and C. Group A engaged in aerobic exercises, Group B participated in meditation, while Group C was administered 20 grams of walnuts daily. Their findings indicated that symptom improvement in the walnut-consuming group was markedly superior compared to the aerobic exercise group, potentially attributable to the high omega-3 fatty acid content in walnuts, which are recognized for their anti-inflammatory and analgesic properties through the inhibition of prostaglandin secretion. They concluded that walnut consumption is more efficacious than aerobic exercise and meditation in alleviating the symptoms of dysmenorrhea. The combination of walnut consumption with aerobic exercises is posited to diminish absenteeism due to sickness and enhance performance among female patients affected by dysmenorrhea [25]. The results of the study conducted by Mohammadi et al. indicated that n-3 polyunsaturated fatty acids (PUFAs) mitigate the severity of primary dysmenorrhea in women, with a meta-regression analysis revealing that daily intake of n-3 PUFAs significantly impacts the severity of primary dysmenorrhea [26]. In our research, baseline measurements of the VAS score, ESR, and CRP values were elevated. However, omega-3 supplementation resulted in a reduction of these parameters across all four groups after 12 weeks of intervention.

CRP levels

Volanakis et al. in their investigation observed that during the acute phase response, which constitutes a defensive mechanism against inflammation, infection, or injury, serum CRP concentrations can escalate up to 1000-fold, attaining peak levels within 24 to 48 hours [27,28]. CRP serves as a crucial biomarker for ongoing inflammatory responses, characterized by a relatively short half-life of approximately 48 hours, with its concentration reverting to baseline levels within seven to 12 days [29]. In our study, the CRP values demonstrated a significant decline across all four groups, achieving statistical significance after 12 weeks, with a p-value of 0.016, indicating a nearly equivalent significant association of CRP values between exercise and omega-3 groups. From the above discussion, it can be noted that exercise along with omega-3 supplementation is beneficial in reducing pain by preventing the secretion of prostaglandins. Consumption of omega-3 supplements along with aerobic exercises can increase performance and decrease the intensity of pain in female patients suffering from dysmenorrhea.

Limitations

This study has some limitations. For example, the mean age of the female patients in the study group was rather low compared to that of the general population. Moreover, since dysmenorrhea tends to decline with age, conducting the study within a specific age group limits its generalizability to the broader population of women.

## Conclusions

This randomized controlled trial investigated the isolated as well as combined effects of regular exercise and omega-3 supplementation on young female patients experiencing dysmenorrhea. The findings suggest that a combined intervention of aerobic exercise and omega-3 supplementation is more effective than either intervention alone in reducing pain and inflammatory markers among young females with primary dysmenorrhea. These findings are noteworthy as the participants experienced a significant decrease in menstrual pain intensity with the combined regimen, underscoring the efficacy of just relying on exercise in alleviating dysmenorrhea-related discomfort. Our study also observed significant reductions in ESR and CRP levels among the participants, which are indicative of systemic inflammation. Their decrease suggests that the interventions have contributed to an anti-inflammatory effect. This aligns with the known anti-inflammatory properties of omega-3 fatty acids, which have been shown to reduce the severity of primary dysmenorrhea. Regular physical activity and omega-3 supplementation are known to influence immune function as well, potentially leading to improved management of dysmenorrhea symptoms. Beyond the physiological benefits, participants reported improvement in overall well-being and daily functioning. Thus, we conclude that the combined interventions not only target the physical symptoms of dysmenorrhea but also contribute positively to the psychological and emotional aspects of health.
